# Advances in 3D printing for the repair of tympanic membrane perforation: a comprehensive review

**DOI:** 10.3389/fbioe.2024.1439499

**Published:** 2024-08-12

**Authors:** Hao Xue, Shengjia Chen, Yi Hu, Juntao Huang, Yi Shen

**Affiliations:** ^1^ Ningbo Institute of Innovation for Combined Medicine and Engineering, The Affiliated Lihuili Hospital of Ningbo University, Ningbo, Zhejiang, China; ^2^ School of Medicine, Ningbo University, Ningbo, Zhejiang, China; ^3^ Centre for Medical Research, Ningbo No.2 Hospital, Ningbo, Zhejiang, China

**Keywords:** tympanic membrane perforation, 3D printing, tissue engineering, repair, wound healing

## Abstract

Tympanic membrane perforation (TMP) is one of the most common conditions in otolaryngology worldwide, and hearing damage caused by inadequate or prolonged healing can be distressing for patients. This article examines the rationale for utilizing three-dimensional (3D) printing to produce scaffolds for repairing TMP, compares the advantages and disadvantages of 3D printed and bioprinted grafts with traditional autologous materials and other tissue engineering materials in TMP repair, and highlights the practical and clinical significance of 3D printing in TMP repair while discussing the current progress and promising future of 3D printing and bioprinting. There is a limited number of reviews specifically dedicated to 3D printing for TMP repair. The majority of reviews offer a general overview of the applications of 3D printing in the broader realm of tissue regeneration, with some mention of TMP repair. Alternatively, they explore the biopolymers, cells, and drug molecules utilized for TMP repair. However, more in-depth analysis is needed on the strategies for selecting bio-inks that integrate biopolymers, cells, and drug molecules for tympanic membrane repair.

## 1 Introduction

The tympanic membrane (TM) is essential for sound conduction. However, trauma, otitis media, and iatrogenic injury can lead to TM perforation (TMP), ranging from minor tears to complete rupture (Anand et al., 2021; Castelhano et al., 2022; Hu et al., 2023). While some perforations heal spontaneously, more considerable injuries often require surgical intervention due to associated otalgia, hearing loss, and patient inconvenience (Anand et al., 2021). The mainstream therapy combines autologous material repair with tympanoplasty (Marchioni et al., 2020; Sainsbury et al., 2022). However, it poses risks such as tissue sampling harm and increased surgical risks, often leading to complications such as foreign body reactions and persistent infections. These conditions highlight the urgent need for moderately priced non-autologous grafts (Sainsbury et al., 2022; Kuo et al., 2018; von Witzleben et al., 2023). Tissue engineering offers repair materials as an alternative to autografts. Bioengineered scaffolds show promise in supporting cell growth, maintaining cell morphology, and promoting extracellular matrix formation (Kuo et al., 2018).

Recent advancements in three-dimensional (3D) printing technology have enabled the fabrication of complex biofunctional patches, allowing precise control over scaffold design and incorporating cells and growth factors to enhance performance (Bracaglia et al., 2017; Kuo et al., 2018; Prendergast and Burdick, 2020). However, selecting appropriate biopolymers and 3D printing methods to restore mechanical properties and sound conduction function remains challenging, given the current state of material and technological development (McMillan et al., 2023; Vyas et al., 2023). Various printing methods, such as inkjet printing, extrusion-based printing, laser-assisted printing, and fused deposition modelling, offer options for fabricating bioengineered scaffolds for TM regeneration (Bozkurt and Karayel, 2021; Kakkad et al., 2023; Brumpt et al., 2024). Meanwhile, combining biological and chemical synthetic materials has led to novel composite materials, including electrospinning membranes, films, and hydrogels, offering potential solutions for TMP repair (Rostam-Alilou et al., 2021).

A comprehensive understanding of 3D printing technology and biopolymers is essential for developing innovative printing inks, selecting suitable 3D printing techniques, and creating tissue engineering strategies. Despite recent articles reviewing TM perforation repair and 3D printing experiments (Aleemardani et al., 2021; Hussain and Pei, 2021; Ilhan et al., 2021; Khan et al., 2022; Vrana et al., 2022; Zhao et al., 2022; McLoughlin S. et al., 2023; Hu et al., 2023; McMillan et al., 2023), only some have examined the specific selection strategies and the role of 3D printing in this context. In this review, we selected studies based on the following criteria: publications from the last 5–7 years, peer-reviewed articles, and studies focusing on the application of 3D printing in tympanic membrane repair. To provide a comprehensive overview, we included several studies that demonstrated the efficacy of 3D printing in TMP repair. For example, Kuo et al. (2018) showed that 3D-printed grafts significantly improved healing rates compared to traditional methods. Similarly, Jang et al. (2022) reported enhanced biocompatibility, versatility, and precision in creating complex, multifunctional scaffolds for tissue engineering using 3D-printed animal models. The subsequent sections will provide additional examples, elaborating on the diverse approaches and promising outcomes in applying 3D-printed scaffolds for tympanic membrane repair.

Despite its promise, 3D printing for tympanic membrane repair faces several challenges, including high costs of materials and equipment, complex production requirements, and time-related drawbacks. Non-professionals require extensive training, and finding suitable bio-ink materials involves significant trial and error. Additionally, long-term clinical studies are necessary to thoroughly assess the risks and benefits of this technology. Moreover, further long-term clinical studies are needed to fully understand the potential risks and benefits of this technology.

This paper aims to fill this gap by extensively exploring the development and innovation of various bioinks and 3D printing technologies, along with their selection criteria, efficacy, and limitations for 3D printing of the TM. In addition, it discusses *in vitro* and *in vivo* trials related to the fabrication of scaffolds for TM repair using these methodologies. This review is structured as follows: Section 1 discusses the current state of TMP repair techniques. Section 2 explores the general overview of 3D printing technologies in TMP repair. Section 3 focuses on the integration of biopolymers, cells, and drug molecules into bio-inks. Section 4 lists the biopolymers available for printing materials used in TMP repair, and Section 5 provides an analysis of future directions and clinical applications.

## 2 Tympanic membrane wound healing

### 2.1 Tympanic membrane structures and compositions

**FIGURE 1:**
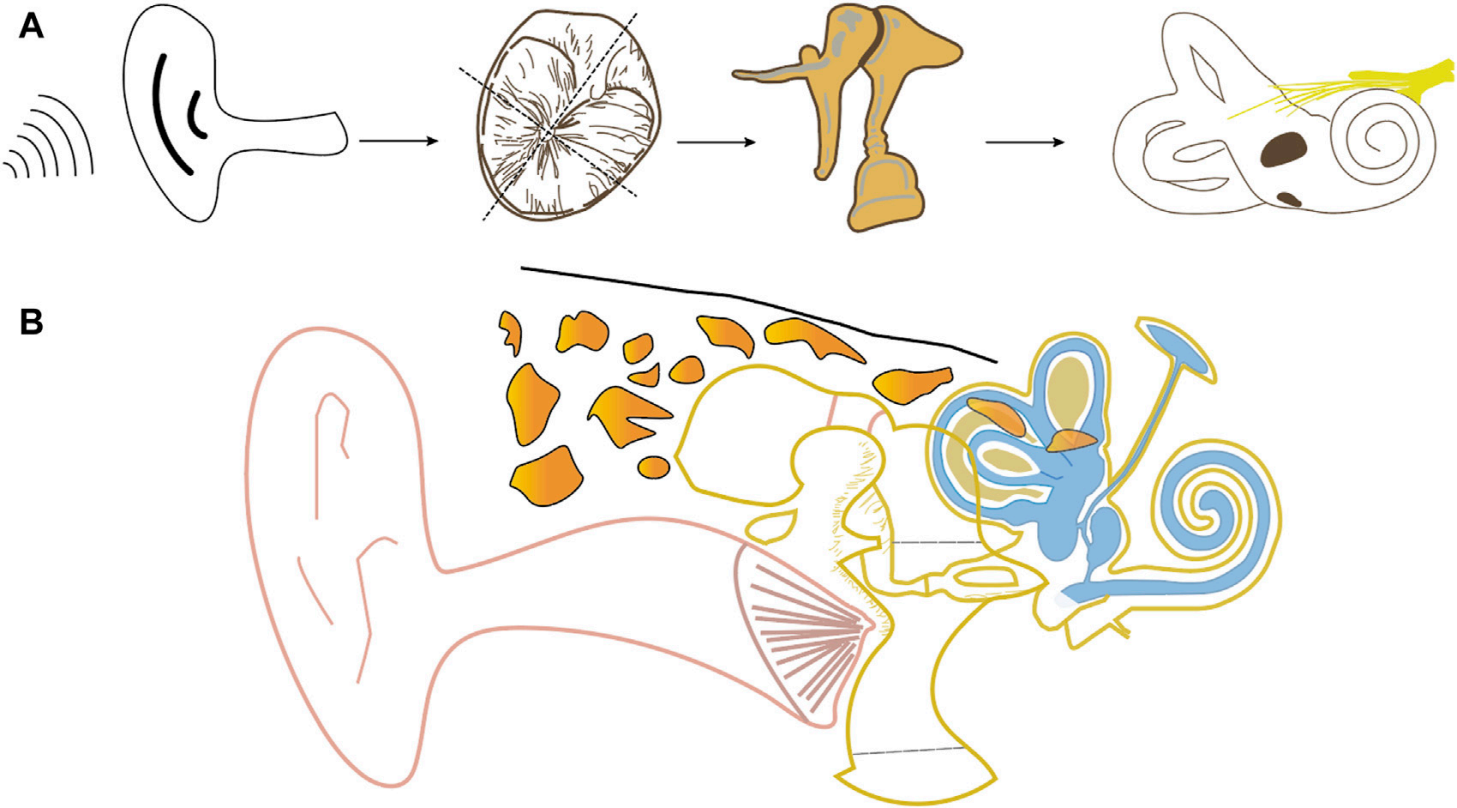
Schematic diagram of frontal anatomy of the ear **(B)** and airborne transmission of sound **(A)** (using the right ear as an example).

The TM serves multiple functions, including insulating the middle ear from external pathogens, maintaining negative pressure, and converting external sound into vibrational frequencies for hearing (Maharajan et al., 2020) (Figure 1). Comprising three layers—outer epithelial, intermediate fibrous connective tissue, and inner mucosa—the TM undergoes continuous regeneration and self-cleaning (Maharajan et al., 2020; Wu et al., 2021). The fibrous connective tissue layer, with its intricate arrangement of fibres, plays a crucial role in acoustic-mechanical conversion and conduction. Various fibre orientations, such as circumferential, radial, and parabolic, influence the TM’s elasticity and resilience. Radial fibres significantly impact rigidity, while circular fibres affect resilience and structural integrity (Anand et al., 2021; Wu et al., 2021).

### 2.2 Tympanic membrane perforation

TM perforations can be classified by their size and location. Minor to medium-sized TM perforations often heal spontaneously or with antibiotic therapy, whereas larger or central TM perforations may become chronic if left untreated (Castelhano et al., 2022; von Witzleben et al., 2023; Ilhan et al., 2021). Persistent perforations, especially those associated with otitis media, can result in hearing loss due to excessive scar tissue or an unstable neo-tympanum. Consequently, it could lead to permanent hearing impairment, especially high-frequency sounds (von Witzleben et al., 2023). Untreated or recurring perforations may also contribute to psychological issues and higher mortality rates, posing significant healthcare challenges.

### 2.3 Current treatments and limitations

Surgical intervention (e.g., myringoplasty) is often necessary for persistent TM perforations (Kuo et al., 2018; Jang et al., 2022). Myringoplasty procedures (type I tympanoplasty) involve placing repair material to elevate the TM flap. The success rate of cartilage repair surgery varies widely, underscoring the importance of surgeon skill in addressing irregular perforation shapes (Kuo et al., 2018). However, reliance on autografts can prolong surgical time and exacerbate postoperative recovery. While exploring using 3D printed guides for autograft fitting shows promise, it remains labour-intensive. Although tissue engineering of the TM is still in its early stages of development, creating pre-prepared uniform repair materials could improve surgical outcomes and accessibility, providing a potentially superior alternative (Rostam-Alilou et al., 2021).

## 3 Strategies of 3D printing

3D printing, as a manufacturing technology, involves the layer-by-layer deposition of ink in the form of droplets or continuous filaments under the influence of mechanical, piezoelectric, electrostatic, thermal, or ultrasonic forces, with each layer accumulating according to predetermined parameters (Liashenko et al., 2020; Dou et al., 2021). These inks may contain natural or synthetic polymers, biological tissues, or a combination (Shahverdi et al., 2022). Considering the diverse regenerative capacities of patients, 3D printing allows for the customisation of bio-ink composition to create patient-specific materials for tympanic membrane repair. Producers can tailor these materials to have anti-inflammatory, antibacterial, and regeneration-inducing properties (Liaw and Guvendiren, 2017; Kuo et al., 2018; Liashenko et al., 2020; Yang et al., 2021). 3D printing-based fibre extrusion technology holds promise in restoring the fibre arrangement of the TM’s connective tissue layer, which is crucial for guiding the arrangement of epithelial cells and fibroblasts, as well as collagen deposition (Anand et al., 2021).

The average thickness of the TM is approximately 0.1 mm (Ilhan et al., 2021; Lee E. et al., 2022; Morgenstern et al., 2024). The development of TM tissue engineering could be enhanced using printing nozzles with adequate precision to fabricate patches. There is more than one printing method capable of achieving sub-100-micron accuracy. The key lies in preserving the loaded drugs’ activity and the cells’ functionality within the product. Currently, stereolithography is more advanced in research on nanoscale printing resolution, achieving a minimum resolution of 65 nm (Hengsteler et al., 2021). However, it has high demands on the ink and can only print photopolymer materials (Hengsteler et al., 2021). 3D printing can systematically and precisely arrange various ink components into three-dimensional engineered structures, offering significant advantages in dimensions, shapes, repeatability, and positional accuracy (Ma et al., 2024; Morón-Conejo et al., 2024). Composite scaffolds made of different polymers and biological components have already been created with the current achievable resolution and have demonstrated promising application outcomes (Brandl et al., 2024; Lipkowitz et al., 2024; Ventisette et al., 2024). Thus, within the current resolution limitations, investigating various bioink components to improve the biological functionality of scaffolds represents a promising research avenue. Therefore, in the following section on 3D printing strategies, it is crucial to discuss integrating biologically active components and cells rather than modified nozzle size. 3D printing infusing bioactive molecules and cells in ink, called bioprinting, enhances cell regeneration potential (Vrana et al., 2022).

The transition of printed products from a fluid to a solid state typically requires crosslinkers or catalysts. Recently, a technology known as 4D printing has emerged, allowing for altering printed product shapes over time dimensions. However, this technology requires materials responsive to external stimuli, which must be utilised (Chen et al., 2023; Pourmasoumi et al., 2023). While no single 3D printing technology can replicate all tissue complexities, inkjet printing, laser-assisted printing, and extrusion printing offer unique advantages, disadvantages, and limitations.

### 3.1 Construct design

Constructing the 3D printed model involves converting image patterns into STL format files, followed by slicing to generate G-code files for the bioprinter tool path. Alternatively, designers can use morphological data or clinical images, such as MRI and CT scans, to create STL files (Boulenger de Hauteclocque et al., 2023; Takaoka et al., 2024; Willershausen et al., 2024). Exploring different toolkit options for creating customised TM models is also feasible (Jiménez et al., 2019). Printer resolution referred to as layer thickness inversely affects print quality and printing time. Porous structures can be achieved by freeze-drying a mixture of solution and emulsifier and removing ice crystals, providing control over scaffold porosity for growth (Abbasi et al., 2020).

CAD techniques are used to acquire patient-specific TM structures, often by scanning the intact TM of the opposite ear to create a CT image for CAD modelling (Bozkurt and Karayel, 2021). Clinical images could offer personalised TM tissue structure information as potential templates for patch printing on-demand. Virtual calibration using clinical image data helps improve resource efficiency. Bio-CAM (Bio-computer-assisted manufacturing) simulates physical models on computers to predict manufacturing feasibility. It often utilises classical formulas and finite element method (FEM) calculations, with the laminar multiphase flow model being widely employed (Amorfini et al., 2018; Mangano et al., 2022).

Studies have extensively investigated the factors affecting cell sedimentation in inkjet printing, including clogging, viscosity, and printing height. Extrusion printing parameters, such as dispensing pressure, printing time, and nozzle diameter, have also been analysed. Laser-assisted printing research has focused on studying the impact of laser energy, base film thickness, and hydrogel viscosity on droplet size, cell proliferation, differentiation, and viability.

Bio-CAM, in conjunction with Bio-CAD, improves print quality and speeds up printing processes, thereby advancing the development of bioprinting technologies. Machine learning algorithms show promise in predictive modelling and parameter tuning for the long-term utilisation of 3D-printed structures.

### 3.2 Printing methods

**FIGURE 2:**
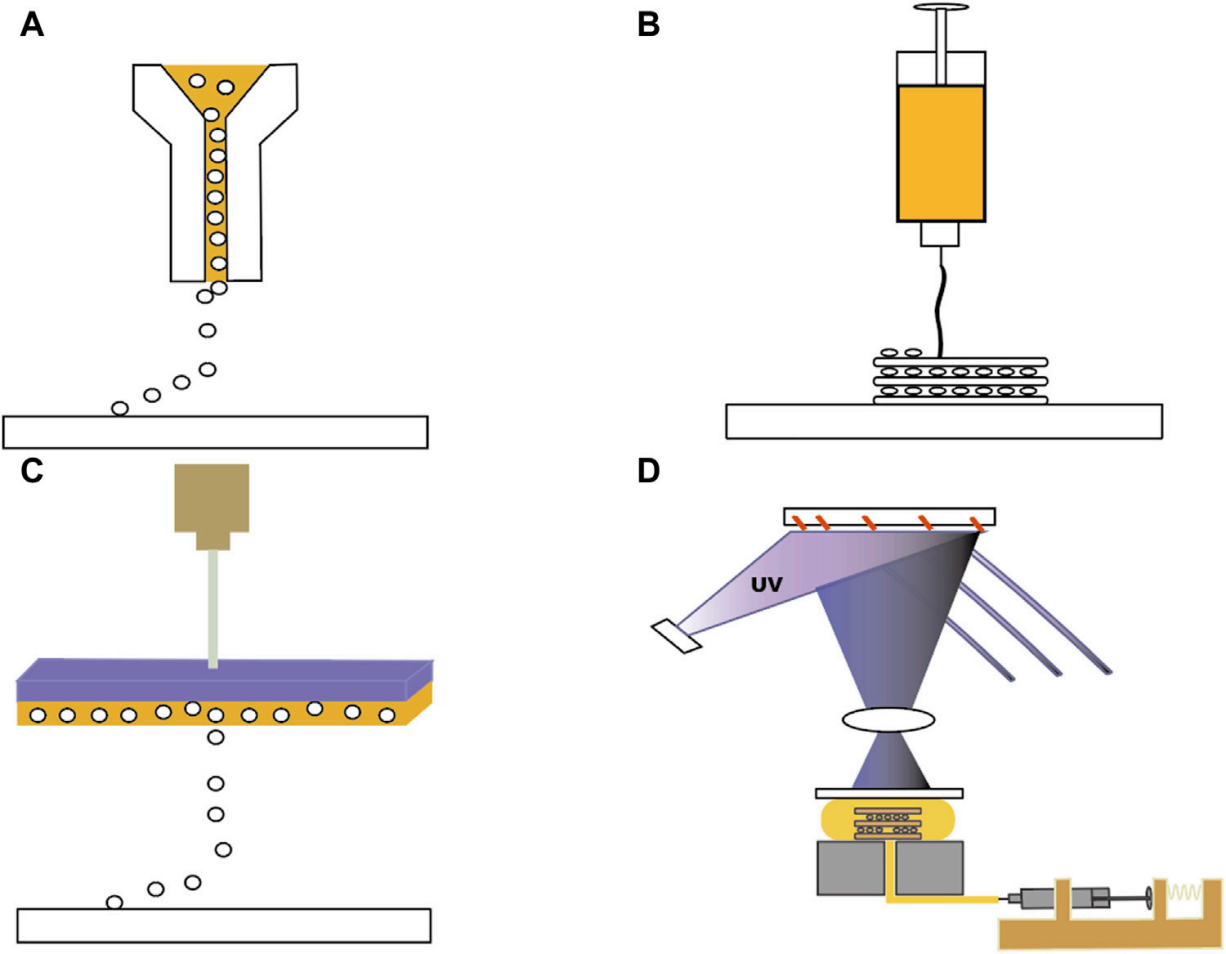
Four different 3D printing methods. **(A)** Inkjet Bioprinting. **(B)** Extrusion Bioprinting. **(C)** Laser-assisted Bioprinting. **(D)** Stereolithography Bio-printing.

When it comes to the printing method, there are several options available (Figure 2), including inkjet printing (e.g., droplet-on-demand, continuous inkjet bioprinting), extrusion-based printing (EBP) (e.g., pneumatic, mechanical, piston, or rotating screw-driven extrusion), electro-assisted bioprinting, light-assisted (e.g., digital light processing), and laser-assisted printing (e.g., laser-induced forward motion, laser-guided direct writing, two-photon polymerisation) to choose from (Ng et al., 2019; Bozkurt and Karayel, 2021; Garcia-Villen et al., 2023).

Inkjet-based 3D printing utilizes nozzle spraying similar to 2D inkjet printing. It offers cost-effective and rapid printing with high cell survival rates. However, it has uneven droplet sizes, necessitating further development of droplet control technology. There is also a possibility that traditional inkjet printing generates thermal and mechanical stress, which can alter cell phenotypes. Therefore, researchers should decrease the cell density and utilize a bioink with low viscosity (Ablanedo Morales et al., 2023; Sörgel et al., 2023). In the field of bioprinting, technological advancements and innovations are progressing rapidly. A new method for gel-free cell printing on Gelatin Methacrylate (GelMA) coated slides has recently emerged. Masaeli and Marquette (2019) applied inkjet printing principles to directly print gel-free cells onto GelMA-coated coverslips, with the aim of creating intricate multilayer cellular models suitable for soft tissue engineering. The research team used a piezoelectric inkjet bioprinter with an 80 μm nozzle diameter and a droplet deposition precision of 5 μm. Despite some variability in droplet diffusion, the spacing between cell droplets remained approximately 210 μm. The absence of hydrogel in the cell suspension resulted in a low viscosity of 1.00E-03 Pa·s, achieving a high cell density of 2.3 × 10^7^ cells/mL. There was no cell clogging in the print nozzle, and this method reduced cell damage compared to traditional inkjet bioprinting. Although repairing TMPs requires an adhesive patch, and the non-adhesive cell model cannot be directly applied, the precision and cell viability highlighted in this study provide inspiration for cell therapies for TMPs. Future research may focus on developing high cell density inkjet-printed scaffolds. Most research and development efforts are directed towards the latter few printing technologies.

EBP shares similarities with inkjet printing but employs higher pressures for viscous inks without high-temperature requirements. It typically uses pneumatic pumps, pistons, or mechanical screws (Vrana et al., 2022). Black (2020) explored the optimal parameters for obtaining isotropic tympanic membrane grafts using a pneumatic extrusion printer for Hot Direct Ink Writing. Jang et al. (2017) detailed the use of EBP and melt-spun in the fabrication of tympanic membrane scaffolds, noting the differences in tissue integration and mechanical properties. The team plotted the MSCs-laden bioink on the PCL/collagen fibrous surface at a processing temperature of 32°C, a pneumatic pressure of 170 ± 15 kPa, and a nozzle moving speed of 10 ± 2 mm/s. High cell density is a fundamental research topic in bio-manufacturing engineering and science, crucial for ensuring key characteristics of newly formed tissues (Dou et al., 2021). EBP, extensively employed in tissue engineering, presents a trade-off between printing precision and maintaining high cell viability at 10^7^ cells per millilitre density (Wang et al., 2022) and could be a popular choice for 3D printing of biomimetic eardrums.

Fused deposition modelling (FDM) combined with electrostatic spinning produces finer fibres and mitigates the drawbacks of traditional electrostatic spinning. However, this method is constrained to creating structures smaller than 3 mm due to charge interactions. Recent studies have combined Fused Deposition Modeling (FDM) with Melt Electrowriting (MEW) and Gel Plotting techniques to fabricate TM patches for perforation repair. This innovative approach explores FDM’s potential in TM patch applications. The hybrid method utilizes FDM’s precision and structural control, while MEW enables the production of ultrafine fibrous networks to replicate the intricate structure of the native TM. Gel Plotting is used to create a pressure-tight membrane by coating the meshes with collagen type I. The study revealed that the conical shape of the patch significantly improved its acoustic properties compared to fiber alignment, which is crucial for sound transmission post-TM repair. This finding contrasts with previous studies on flat patches. The research emphasizes the importance of achieving a conical structure in TM patches to mimic the native TM accurately and highlights the advantages of 3D printing in producing such structures with precision. Despite FDM’s benefits in controlling macro-architecture and ensuring mechanical strength, challenges remain in bioprinting to replicate the native TM and in substituting PCL with more biocompatible materials.

Furthermore, the need for post-processing to remove support materials can complicate the production process. Xie and colleagues (Xie et al., 2019) investigated an electrically assisted bioprinting method. Instead of using the commonly employed PCL material in FDM, the researchers utilized GelMA loaded with cells for printing. Unlike FDM, which typically uses PCL, this study employed cell-laden GelMA for printing. They initially fabricated low-concentration GelMA hydrogel microspheres loaded with bone marrow stem cells. By utilizing the inherent electric force, the method ensured the uniform formation of droplets ejected from the nozzle without dispersion or shape distortion while retaining the cellular capabilities of the loaded cells. They further confirmed the effectiveness of drug loading by producing microdroplets containing dextran and fluorescein. Currently, the application of FDM in TM repair is limited. Combining these technologies and materials shows promise as an advancement in developing effective TM repair patches.

Laser-assisted printing techniques, such as digital light processing and stereolithography, utilise laser pulses to activate donor layers (Zhou X. et al., 2024; Mohammad Mehdipour et al., 2024). Digital light processing is an enhanced version of stereolithography that enables faster production (Yilmaz et al., 2024). The platforms offer advantages such as non-porous printing, superior biocompatibility, high resolution, efficiency, and smoother interlayer interfaces (Cao et al., 2022). Nevertheless, their current limitation lies in the ability to print materials suitable for photo-polymerisation, necessitating additional steps for chemical modification of the ink (Aksit et al., 2018). Furthermore, the need for ink to fill the reservoir raises concerns about material wastage, especially for cell-laden and active molecule-laden materials, which may result in higher costs. Research on utilizing this technology for TM patches is still under investigation. Nobus (Nobus et al., 2024) and colleagues developed three types of scaffolds using digital light processing: Norbornene-modified gelatin (GelNBNB), Gelatin methacryloyl (GelMA), and alkene-functionalized PCL (E-PCL). Stereolithography requires pre-printing sample testing using UV-VIS spectrophotometry, droplet tests to determine effective resin crosslinking parameters, and rheology and viscosity testing to ensure successful scaffold fabrication. While these scaffolds exhibited good physicochemical properties and cell compatibility, their performance in repairing acute TM perforations in a rabbit model was suboptimal, with some perforations remaining unhealed 4 weeks post-surgery. The scaffolds’ low content of biopolymers and other active components may not adequately induce tissue repair. Further animal and *in vitro* studies are needed to refine the ink formulations to enhance repair efficacy.

Combination printing methods, such as coaxial printing and extrusion combined with electrospinning, provide innovative approaches for high-resolution printing and scaffold stability, offering potential applications in TM tissue engineering. A study conducted by Chen et al. represents a groundbreaking effort in producing gelatin/poly (lactic-co-glycolic acid) (PLGA) electrospun fibres for three-dimensional (3D) printing. This process results in customised scaffolds with controllable shapes and large pores (Chen et al., 2019). By integrating spiral-assisted additive manufacturing and rotational electrospinning techniques, researchers developed multi-layered polymer/glass scaffolds characterised by hierarchical porosity, high mechanical strength, controlled degradation, and excellent biocompatibility (Touré et al., 2020). Another innovative approach that combines 3D printing and electrospinning technologies has facilitated the production of dual-scale anisotropic scaffolds. This approach offers a promising avenue for advancing musculoskeletal tissue engineering and addressing the challenges of treating considerable bone defects (Huang et al., 2020).

The TM is a specialized membrane structure that needs to be constantly mobile. Perforations in the TM usually occur in the pars tensa, and perforations larger than 25% are critical points that affect its motion and sound transmission functions (Stomackin et al., 2019). The microstructure of the TM is closely related to its acoustic properties. The mouse tympanic membrane is commonly used as an animal model in hearing research because it exhibits low-frequency hearing characteristics similar to humans (Stomackin et al., 2019). Its collagen fibres in the outer layer of the pars tensa are radially oriented, while in the inner layer, they are circumferentially oriented and interwoven. Elastic fibres are distributed in radial and circumferential directions, coexisting with collagen fibres, spindle fibroblasts, the capillary network, and vimentin-positive cells that grow within this matrix. No differences in the directional distribution of elastic fibres have been found (Wu et al., 2021). The discovery of this 3D structure inspires the material arrangement in printed TM patches, potentially enhancing the ability of additive-manufactured patches to accommodate hearing compensation during the TM healing process, thus highlighting the structural controllability advantage of 3D printing.

Many existing multi-material 3D printers can meet the demand of printing a product with different ink components simultaneously, suggesting the feasibility of depositing various fibres separately. For example, Brown et al. (2022) utilized the multi-material printer Objet350 Connex3 3D printer (Stratasys, Eden Prairie, MN) to design and print a blast test model of the ear that combines hard tissue (temporal bone) and soft tissue (external auditory canal, TM). The pressure changes in this model during blast testing were similar to those in human temporal bone donors, demonstrating the effectiveness of this type of 3D-printed anatomically accurate model.

These achievements are all thanks to more intelligent printing machines, continuously improved printing techniques, and optimized printing parameters. They indicate the robust development and promising prospects of 3D printing technology, providing a solid technical foundation for manufacturing 3D-printed patches for repairing TM perforations.

### 3.3 Applications in other organs

The integration of two or more technologies to fabricate regenerative scaffolds has been successfully demonstrated in various fields, showing promising prospects and feasibility for application in tissue engineering. Moreover, the techniques already utilized in other tissues offer valuable insights for experimental exploration and research into TM repair.

Drawing insights from the advancements in 3D printing applied to other tissues is of significant value for assessing the prospects of 3D printing for the TM. For instance, in bone and cartilage tissue engineering, various 3D printing techniques, such as inkjet printing, extrusion, stereolithography, and selective laser sintering, are commonly employed (Feng et al., 2021; van der Heide et al., 2022). However, current methods primarily focus on powder deposition rather than achieving a natural, smooth interface. To address this issue, researchers are investigating utilising fully elastic materials and integrating nanomaterials such as graphene into stress-relaxed hydrogels or chemically modifying hydrogels. This approach holds promise for the development of composite materials.

Furthermore, advancements in soft tissue additive manufacturing, including cell-based printing for tissues such as cartilage, cornea, heart, hair follicle, retina, skin, and liver, have seen significant progress (Masri et al., 2022; Ainsworth et al., 2023; Fang et al., 2023; Kang et al., 2023; Maihemuti et al., 2023; Son et al., 2023; Xu et al., 2023). In particular, skin tissue engineering has initiated *in vivo* bioprinting trials. Inspired by layered printing techniques and the incorporation of drugs and biologically active agents into scaffolds, researchers are exploring innovative approaches for tissue engineering applications.

In retinal fabrication, 3D printing technology offers numerous benefits in tissue engineering and regenerative medicine. Masaeli et al. successfully printed a layered retinal model using a carrier-free bioprinting method with a piezoelectric inkjet dispenser. This method resulted in a higher cell population compared to classical tissue culture plates, showcasing the potential of acellular printing in membrane tissue transplantation (Masaeli et al., 2020). Furthermore, 3D printing technology has significantly advanced thin film manufacturing. Kim et al. (2018) used cultured human corneal endothelial cells (HCECs) engineered to overexpress RNase 5 (R5-HCECs) and collagen as bioinks, incorporating 0.02% arginyl-glycyl-aspartic acid (RGD) to minimize cell loss. They successfully printed a cornea with excellent optical properties, providing new treatment options for ocular diseases. This grid-like soft tissue structure achieved a seven-layer configuration, whereas the TM typically consists of three layers: inner, middle, and outer. The microanatomy suggests that, by utilizing such formulations, a biomimetic TM could be printed by directly including TM epithelial cells and fibroblasts into the bio-ink instead of relying on stem cells that need differentiation. This method has the potential to produce a microstructure closely resembling the native TM, thereby improving the effectiveness of patch-induced TM structural remodeling.

The confocal structure of the tympanic membrane (TM) presents a unique challenge in its replication compared to other tissues. Traditional 3D printing typically involves additive manufacturing from the bottom to the top layer on a flat platform, resulting in products that do not inherently form a conical structure. However, advancements in retinal tissue engineering have already led to the development of dome-shaped or convex corneas. For example, Xu et al. (2023) ingeniously utilized the gravity of droplets and the thermo-responsive nature of the bioink to design a smooth convex structure closely resembling the natural cornea. The tip thickness of this structure could vary with the printing temperature and altering the GelMA/collagen ratio in the ink could adjust its transparency and mechanical properties. While the parameters in their study were optimized for corneal biomimetics and thus are not listed here, this concept inspires the potential fabrication of a conical, transparent TM patch by adjusting similar parameters.

In Campos’s laboratory (Duarte Campos et al., 2019), type I collagen and agarose hydrogel were used as bioinks with a 300-micron nozzle equipped with an electromagnetic micro-valve, employing a drop-on-demand (DoD) bioprinting strategy instead of micro-extrusion printing. By evenly distributing and depositing four types of droplets in a specific spatial sequence, they successfully fabricated a dome-shaped biomimetic corneal structure, with human corneal stromal keratocytes demonstrating viability comparable to that in the native cornea. However, these innovative ophthalmic studies require further modifications, such as scaling down product specifications and increasing adhesion, before being applied to the TM. The potential to replicate the native shape of the tympanic membrane indicates a promising future for 3D printing in developing biomimetic tympanic membranes, highlighting the promising future of 3D printing in creating biomimetic TM grafts.

## 4 Bioink properties and tissue engineering advances

The formulation of 3D printing ink for tissue engineering involves liquid biopolymers, many of which are absorbable and commonly used in tissue healing and regeneration (Yeleswarapu et al., 2023). Designing bioink formulations is one of the critical and complex steps in 3D printing. Sometimes, single-material inks fail to meet the dual viscosity requirements for 3D printing and compatibility for tissue degradation (Navara et al., 2023). Blends and composites of different materials have emerged as viable alternatives, particularly in various human tissue 3D printing applications such as TM patching. This section examines the desirable properties of hydrogels and highlights novel crosslinked hydrogels used in tissue engineering, along with an overview of added cells and growth factors. Bioink development has introduced pure cellular printing alongside cell-free and cellular inks (Yeleswarapu et al., 2023). Most bio-inks consist of a hydrogel, prepolymer solution, and cells, with the hydrogel playing a critical role in providing structural support and determining the bio-inks fundamental physical and chemical properties. To repair perforations in the TM, injectable materials are not feasible; it is necessary to print a structure with sufficient mechanical strength to support the membrane’s vibrations at various frequencies. The ideal scaffold would closely replicate the native structure of the tympanic membrane, with bioink filaments arranged to induce cell proliferation and migration in a manner that optimizes sound transmission, gradually degrading over time. This process involves not only the performance parameters of the printing machine but also the indispensable enhancement of resolution through the modification of bioink formulations. Ideally, the hydrogel should be printable, cross-linkable, mechanically robust, biocompatible, and have controllable degradability (Pitzanti et al., 2024).

The relationship between the physicochemical properties of bio-inks and their impact on material printing and tissue growth is intricate and multifaceted. Achieving the optimal solution ratio for bio-inks is crucial for enabling healthy cell and tissue growth while ensuring the appropriate surface tension and wettability of ink droplets (Navara et al., 2023). Proper tension enables precise deposition of droplets and prevents collapse, while wettability influences droplet height and distribution. Viscosity influences cell distribution and scaffold fidelity, but excessive viscosity hampers extrudability (Navara et al., 2023). Shear thinning, where the viscosity decreases with flow rate, is desirable for maintaining cell distribution and extrudability simultaneously.

It is important to note that the tympanic membrane (TM) is a tiny and thin structure, with an average size of 8 mm by 9 mm and a thickness of only 0.1 mm. Repairing such delicate tissue requires equally light and thin materials to avoid damaging the wound edges during the repair process. Thinner grafts demonstrate higher sound-induced velocity for isotropic TM grafts than thicker ones when conducting low-frequency sounds. Therefore, in the context of current extrusion-based 3D printing techniques commonly used for fabricating biomimetic TMs, it is crucial to use nozzles with a small inner diameter and fine ink filaments.

Black (2020) provided optimal parameters for a multi-material hot printhead pneumatic printer, specifying a nozzle inner diameter of 200 µm and a movement speed of 20 mm/s. Using these parameters, they fabricated biomimetic TM grafts with a diameter of 8 mm from polycaprolactone diol (PCL), P-PCL (PCL blended with 25% wt poly (ethylene glycol) (PEG)), poly (ester urethane urea) (PEUU), and P-PEUU (PEUU blended with 25% wt PEG). The researchers arranged the ink filaments in these grafts into 50 concentric circles and 50 radial fibres. Human keratinocyte and fibroblast cells were seeded onto these grafts, showing proliferation on all materials and forming distinct topographical features, with the highest degree of alignment observed on P-PEUU grafts and the lowest on PCL grafts. These patches were degradable by lipase, with P-PEUU grafts showing the highest degradation rate and PCL grafts the lowest. The anisotropic PEUU and P-PEUU grafts developed by this group exhibited adjustable mechanical strength similar to the human TM. By altering the air pressure and printing speed, they balanced the requirements for filament diameter, shear force, and extracellular matrix alignment. The biomimetic materials demonstrated good biocompatibility, suggesting their potential in clinical TM patches that enhance effective sound conduction. This study provides valuable insights into the settings for 3D printing inks in the fabrication of biomimetic TMs, highlighting the potential of 3D printing in TM repair.

Various bioinks, incorporating novel combinations and chemical modifications, address scaffold quality control issues (McLoughlin S. T. et al., 2023). For instance, Sharif et al. developed a glycidyl methacrylate-modified gelatin with enhanced mechanical properties, as demonstrated in corneal repair. Different crosslinking methods, such as ionic, thermal, photo, or enzyme-based methods, offer versatility in bioink properties and 4D printing capabilities. Combining materials cured in different ways allows for temporary shaping during printing and subsequent curing.

Recent advancements include utilising anion-cation charges to alter hydrogel network properties over time, enabling 4D printing with reduced biotoxicity. Additionally, reversible crosslinking methods, such as those involving Ca^2+^ and thermoresponsive chains, offer controllable scaffold properties. Cryogelation methods and photo-cross-linkable baths can enhance scaffold stability and mechanical properties.

Given the abundance of existing research on 3D printing in bone and cartilage, its application in the bone and cartilage reconstruction in the otolaryngology field has been at the forefront. Precedents occurred for regenerating auricular cartilage and the ossicular chain, demonstrating promising outcomes in reconstruction (Zhong and Zhao, 2017). The application of tissue engineering in the external ear and tympanic membrane continually evolves. The external ear must maintain a specific shape, and the tympanic membrane must resist sound wave vibrations; both require scaffolds with sufficient mechanical strength for effective repair. Thus, in preparing bioink for 3D printing the tympanic membrane, it is essential to incorporate compounds that enhance mechanical strength. This requirement distinguishes it from the repair of other membrane-like tissues such as skin, sclera, and cornea. Incorporating stem cells into bio-inks shows promise for tissue repair, but challenges remain in providing a suitable environment for inducing cell differentiation and proliferation.

The tympanic membrane (TM) is a thin tissue layer that connects the external ear canal to the middle ear cavity, with both sides exposed to air. When placed as a patch on the TM, it tends to dry out, which is not conducive to cell growth (Jang et al., 2017). Therefore, a bioink with good swelling properties is more suitable for creating patches for this specialised tissue; given the current advancements in material development, ink in hydrogel form is a promising option. Bioprinted scaffolds, such as polycaprolactone/collagen/alginate-MSC (PCAMSC) scaffolds, provide a moist environment and deliver stem cells, demonstrating effectiveness in TM repair, leading to enhanced healing rates and hearing recovery.

A study compared four different combinations of collagen-based bioprinting scaffolds with human adipose stem cells (hASCs), basic fibroblast growth factor (bFGF), and human-derived umbilical cord serum (hUCS): CEC, CEC-F, CEC-U, and CEC-FU. These combinations were used to assess their effects on cell proliferation and migration (Jang et al., 2022). The results indicate that scaffolds containing growth factors and serum successfully facilitated both the proliferation and migration of keratinocytes. Notably, the scaffold supplemented with hUCS and bFGF exhibited the most significant enhancement in promoting TM regeneration. However, long-term effects on tissue thickness and potential tumour formation require further investigation.

Extruding a qualified cell-laden bioink must be stable under the shear forces exerted during printing, and the extruded material must remain insoluble under physiological conditions. It should closely mimic the extracellular matrix (ECM), possessing excellent biocompatibility and controllable gelation and degradation times (Gupta et al., 2021). Combining biologically active molecules, such as serum and growth factors, with cells and polymer scaffolds demonstrates superior efficacy in promoting TM healing compared to individual components. By modifying the bioink formulation, the cellular distribution and proliferation within cell-laden scaffolds fabricated by bioprinting can surpass those achieved through manual seeding. The collaborative interaction between bioactive ingredients and stem cells in promoting keratinocyte migration and subsequent TM reconstruction highlights the potential efficacy of advanced tissue engineering strategies for addressing TM perforations.

## 5 Biopolymers

The use of biopolymers in fabricating tympanic membrane (TM) grafts has seen significant advancements in recent years. These materials were selected for their unique properties, which include biocompatibility, controllable mechanical characteristics, and the ability to degrade safely within the body (Chuang et al., 2024; Jia et al., 2024; Li et al., 2024; Zhu et al., 2024). This section delves into the rationale behind choosing specific biopolymers for TM repair, highlighting their advantages and disadvantages. Furthermore, we will present case studies illustrating the practical applications and outcomes of using these biopolymers in clinical settings. Through a detailed examination of traditional and more recent materials, such as the shift from conventional polymers to innovative biopolymers like silk, we aim to comprehensively understand the current landscape and future directions in TM graft fabrication.

Gelatin was chosen for its biodegradability and medical use, and the FDA has approved it. As a commonly used bulking agent, it is biocompatible but lacks mechanical solid properties, and uncrosslinked gelatin is typically soluble (Rodriguez et al., 2017). By modifying gelatin with photocrosslinkable methacrylamide groups, the widely used material GelMA turns up. GelMA is combined with other polymers for various printing techniques in tissue engineering due to its excellent biocompatibility. Its controllable physicochemical properties and easily modifiable structure make it more advantageous than other photosensitive materials in light-assisted printing technologies such as stereolithography and digital light processing (Shi et al., 2024; Yilmaz et al., 2024).

One challenge with using low concentrations of GelMA is its low viscosity, making it difficult to maintain shape during printing—a common issue for natural biopolymers. However, hydrogels made by combining GelMA with different materials can overcome this limitation. GelMA can be part of an ink formulation or used alone to create drug-loaded microspheres for hydrogel drug delivery systems (Su et al., 2024). These drug delivery systems can enhance drug bioavailability, reduce systemic side effects, and decrease dosing frequency, making them increasingly common in tissue engineering scaffolds. Biodegradable and biocompatible biopolymers are the preferred materials for drug carriers. Keratin is a structural protein found in hair, nails, and feathers rich in cell-binding sequences promoting cell adhesion and proliferation, making keratin-based hydrogels highly biocompatible. A Laboratory developed an innovative multi-drug combination therapy scaffold, GEN@PVA/GelMA-KerMA, which incorporates antibacterial drug gentamicin (GEN) and growth factor FGF-2-loaded nanoparticles, using GelMA-KerMA as the bioink for 3D printing. The scaffold features conical microneedles on its surface. It treats TMPs by providing sustained local drug release, controlling infections, and promoting epithelial regeneration while maintaining tympanic membrane integrity. This patch exhibits good antibacterial performance against *P. aeruginosa*, *S. aureus*, and *E. coli*. It also demonstrates good biocompatibility, promoting cell attachment and proliferation due to the rough surface of the FGF-2@GEN@PVA/GelMA-KerMA patch and the incorporated drug FGF-2 (Bedir et al., 2024).

Alginate is a widely used material for TM repair, and researchers are designing and printing alginate-based patches. Concerns arise regarding the compatibility of these patches with the irregular perforated edges of the TM and their ability to support tissue growth. The study by Jang and his team successfully addressed this issue. Their previous research pointed out that combining collagen and umbilical cord serum (UCS) can effectively accelerate cell growth and promote the repair of chronic TMPs. In their subsequent experiments, they developed MSC-laden alginate as a bioink, aiming to create a cell-laden scaffold composed of collagen and alginate. Considering the insufficient mechanical strength of alginate and collagen scaffolds, they incorporated polycaprolactone (PCL) fibres to enhance mechanical strength. The cell-laden matrix exhibited high cell viability, reaching 94.1% ± 1.5% (Jang et al., 2017). Another study examined the potential of 15 wt% polylactic acid (PLA) scaffolds mixed with 3 wt% chitosan (CS) or 3 wt% sodium alginate (SA) for repairing TMs.The researchers identified the 3 wt% CS or 3 wt% SA ratios as the most printable. The 3D printing scaffold mimicked the thickness of the natural tympanic membrane, demonstrating successful adhesion and distribution of mesenchymal stem cells. The *in vitro* results indicate their suitability for personalised, cost-effective tissue repair patches. Both scaffolds exhibited enhanced swelling properties, which indirectly increased material elasticity, helping the scaffold to adapt to the vibrations during the healing process of the tympanic membrane without dislocation or rupture, further confirming the compatibility of alginate and other biopolymers with 3D-printed biomimetic tympanic membranes (Ilhan et al., 2021). Despite demonstrating the feasibility of a cell-friendly extrusion printing system, further *in vivo* experiments are necessary to validate its efficacy.

As listed in Table 1 under Bioink Ingredients, collagen is frequently used as a component of bioink in recent 3D-printed tympanic membrane (TM) scaffolds. Collagen, a natural extracellular matrix component, contains functional peptide chains that promote cell adhesion, proliferation, and differentiation, exhibiting excellent biodegradability and low immunogenicity (Nayak et al., 2023; Chen et al., 2024). The orientation of natural collagen fibres varies across different human tissues, providing an inductive environment for cell proliferation and differentiation. Scaffolds containing oriented collagen can enhance the mechanical properties of load-bearing tissues, and collagen-based bioink, neutralised with TRIS-HCl, offers a milder cell printing environment (Garcia-Villen et al., 2023; Zhou Y. et al., 2024). Although collagen is abundantly sourced and relatively easy to obtain, the stringent conditions required for collagen extraction pose challenges in ensuring consistent quality across batches (Garcia-Villen et al., 2023). Another limitation is its softness and low viscosity, resulting in low resolution and insufficient mechanical strength when printed as a scaffold alone (Montalbano et al., 2023).

**TABLE 1:** 3D applications in otology.

Scaffold structure	Bioink ingredients	Printing methods	Model	Outcome evaluations	References
Circumferential and radial filament	Polydimethylsiloxane (PDMS)/flex-polylactic acid (PLA)/polycaprolactone (PCL)/fibrin-collagen composite hydrogel	Digital optoelectronic holography 71 (DOEH)	*In vitro*	The motion at lower frequencies approximates that of the human tympanic membrane, and the motion at higher frequencies is superior to that of the temporalis fascia. The greater the number of fibres in the scaffold, the higher its mechanical load-bearing capacity, with no decrease in elasticity	Black (2020)
Rectilinear infilling pattern with pores	Polylactic acid (PLA)/chitosan (CS) or sodium alginate (SA)/sodium hydroxide solution/calcium chloride solution	Extrusion printing	In vitro	The scaffolds show an increased swelling ratio and good biocompatibility, improving the cell viability and the mesenchymal stem showed an excellent attachment to it.	Ilhan et al. (2021)
Biomimetic P-PEUU 50Circumferential/50Radial filaments graft with a diameter of 8 mm	Poly (ethylene glycol) (PEG)/poly (ester urethane urea) (PEUU)	Hot direct ink writing (HOT-DIW)	Chinchillas	The native tissue grows on both the medial and lateral sides of the graft, with arranged collagen fibres being deposited indicates that native cells can remodel the biodegradable material into native tissue, the patch exhibits the the highest rate of successful tympanoplasty than fascia and Biodesign® grafts	Black (2020)
Porous scaffold	Norbornene-modified gelatin (GelNBNB) or Gelatin methacryloyl (GelMA) or alkene-functionalised PCL (E-PCL)	Digital light processing (DLP)	*In vitro* and *in vivo* (female adult rabbits) and *ex vivo*	The E-PCL prints had the lowest swelling degree and the highest storage modulus, whereas the norbornene-modified gelatin (GelNBNB) was of the opposite trend. All three scaffolds were biocompatible until day 28. E-PCL scaffolds induced the most efficient healing out of the three scaffolds. More in vivo and *ex vivo* tests were needed	Nobus et al. (2024)
Funnel-shaped membrane with grid or radial/circular filaments	Polycaprolactone (PCL)/collagen type I	Fused deposition modelling (FDM), gel plotting, and melt elec Trowriting and sacrificial structure	*In vitro*	The acoustic properties of the conical scaffolds were close to native TM, which induced faster cell coverage	von Witzleben et al. (2023)
A nanoparticle-coated 3D-printed hydrogel patches containing conical microneedles with a coaxial coat	Photocrosslinkable gelatin methacryloyl (GelMA) /keratin methacryloyl (KerMA)/PVA nanoparticles /gentamicin (GEN) /fibroblast growth factor (FGF-2)	Digital light processing (DLP)	*In vitro*	The patch exhibits good antibacterial performance against *P. aeruginosa*, *S. aureus*, and *E. coli*. Regarding its impact on cell viability, it demonstrates good biocompatibility, promoting cell attachment and proliferation	Bedir et al. (2024)
A support structure and the sensor	Bisphenol-A ethoxylate methacrylate (BEMA)/BaTiO3 500 nm nanoparticles (NPs)	Digital light processing stereolithography (DLP-SLA)	*In vitro*	The acoustic response phenomena between the 3D-printed sensor and the locust tympanic membrane are similar for each frequency	Domingo-Roca et al. (2018)
Epithelium and stroma mimicking structures	Human embryonic stem cell-derived limbal epithelial Stem cells (hESC-LESC)/human adipose tissue-derived stem cells (hASCs) /recombinant human laminin/human sourced collagen I	Laser-assisted Bioprinting (LaBP)	*In vitro*	The biomimetic structure demonstrated robust cell survival and differentiation within the porcine corneal organ	Sorkio et al. (2018)
Tympanic repairing scaffolds	Polycaprolactone/collagen/alginate-mesenchymal stem cell (PCAMSC)	Extrusion bioprinting	Sprague-Dawley Rat (SD-Rat)	The experimental group’s closure rate of perforation exceeded that of the control group, and superior recovery of ABR thresholds and regenerated tympanic membrane thickness was observed across all frequencies in the experimental group compared to the control group	Jang et al. (2017)
Customised 3D-printed guiding template	Gelatin sponge particles	Inkjet printing	Human	The template markedly reduced the operation time, enhanced the closure rate, and decreased the postoperative air-bone gap (ABG)	Yang et al. (2021)
Butterfly-structured tympanic repairing grafts	GelMA/2‐hydroxy‐1‐(4‐(hydroxyethoxy)phenyl)‐2‐methyl‐1‐propanone (Irgacure 2959; BASF)/Fibronectin (FN)/epidermal growth factor (EGF)/Murine fibroblast cell line NIH/3T3	3D Bioprinting	female chinchillas (Chinchilla lanigera)	The butterfly structure eliminates the need for surgical glues or sutures, enhances mechanical stability, and accelerates wound healing, improving the healing of tympanic membrane perforations	Kuo et al. (2018)
mesh-structure scaffolds	Human-derived umbilical cord Serum (hUCS)/Collagen/Basic fibroblast growth factor (bFGF)/Human adipose stem cells (hASCs)	Extrusion printing	Sprague-Dawley Rat (SD-Rat)	It accelerated cell proliferation and induced keratinocyte proliferation, promoting TMP regeneration	Jang et al. (2022)

Hyaluronic acid, also derived from the extracellular matrix, is a commercially available natural polysaccharide commonly used in transdermal drug delivery systems. It possesses excellent moisture retention capabilities, providing a humid environment conducive to cell growth. However, its complex extraction process, low mechanical strength, and potential limitations on cell proliferation and differentiation are notable drawbacks. Hydrogels with good swelling properties can similarly offer a moist environment for cells (Wang et al., 2023; Nita et al., 2024). Although there are few cases of 3D printing TM patches with hyaluronic acid, its transdermal drug delivery characteristic might provide new insights into developing TM drug-releasing patches.

A single biomaterial, such as hyaluronic acid (HA) or collagen, may have low mechanical strength and short gelation times, making it unsuitable as a standalone printing ink. Mixing multiple materials in appropriate ratios can address these issues, enhancing printability, structural stability, and controllable degradation rates of the bioink. Optimal combinations can also mitigate the limitations posed by toxic crosslinkers and high-temperature curing in bioprinting (Duarte Campos et al., 2019; Moro et al., 2022). For instance, methacrylated hyaluronic acid (HAMA), obtained by modifying hyaluronic acid, is suitable for stereolithography printing, producing hydrogels with excellent stiffness and cell viability (Mohammad Mehdipour et al., 2024).

Silk fibroin contains various reactive groups and residues that can undergo covalent crosslinking. The transformation from random coil to β-sheet crystallisation induces a sol-to-gel transition, making silk fibroin an ideal structural matrix due to its cytocompatible crosslinking methods, ease of procurement and processing, controllable degradability, and mechanical properties (Sun M. et al., 2021). Degummed natural silk has been approved by the Food and Drug Administration (FDA). It can be modified through extraction, blending, grafting polymerisation, self-assembly, crosslinking, interpenetrating network, and enzymatic catalysis (Xu et al., 2024). Studies have demonstrated that silk can be non-toxically crosslinked with glycerol, resulting in scaffolds with good structural stability. These scaffolds, tested *in vivo* in mouse models, maintained their structure for up to three months—significantly longer than the 30 days typically required for chronic TMP repair. This characteristic, combined with its controlled complete biodegradability and minimal inflammatory response, highlights the advantages of silk-based bio-inks in TMP repair (Rodriguez et al., 2017). Another combination of silk and gelatin has been applied to repair rat skin defects. This scaffold, incorporating fibroblast growth factor 2 (FGF-2) into gelatin-sulfonated silk, supported cell growth and promoted angiogenesis, addressing clinical challenges in full-thickness skin defect repair (Qi et al., 2017). Combining silk with other biopolymers, such as gelatin and collagen, is a common strategy to enhance the performance of these bioinks (Huh et al., 2018; Li X. H. et al., 2021; Li Q. et al., 2021). Applying this to the tympanic membrane, which also faces issues of fibrous overgrowth in traditional repairs, suggests the potential for 3D-printed porous silk-based scaffolds with additional growth factors to test their ability to restore the TM’s three-layer structure to its native state.

Furthermore, regenerated silk in composite scaffolds helps enhance cell adhesion, differentiation, and proliferation, showing promising potential in tissue regeneration (Kumari et al., 2020; Sun W. et al., 2021). A unique feature of silk fibroin, compared to other biopolymers, is its ability to undergo sol-gel transitions in response to enzymes or sound waves without requiring high-temperature or acidic environments for curing, which helps maintain cell viability in cell-laden inks and control gelling conditions (Fu et al., 2022). When used as load-bearing materials, natural silk requires sericin removal to reduce molecular weight, which can affect bulk viscosity and degradation ratios (Ealla et al., 2022). Although the TM is not a load-bearing tissue, it must vibrate in response to sound waves. Thus, the mechanical strength of sericin-free silk fibroin patches must be further tailored and studied to meet the demands of different batches. The degumming process may present a technical challenge in interdisciplinary efforts to fabricate biomimetic TM patches (Sun W. et al., 2021)^.^

Currently, extrusion printing is the predominant method for 3D printing silk, but silk protein structures can also be modified to suit other printing techniques. For instance, researchers have developed a bioink by covalently crosslinking fluorescent silk fibroin with glycidyl methacrylate (GMA) and successfully used digital light processing to print an outer ear model (Lee Y. J. et al., 2022)^.^ Although the application of silk-based bioink in TM repair is still unexplored, other laboratories have achieved promising results using silk to fabricate TM patches. For example, a study used electrospinning to create patches from polycaprolactone (PCL) and silk blends and tested their application in smaller TM perforations. These patches adhered firmly to the repair site with minimal fluid from the temporal bone, aiding TM healing (Benecke et al., 2022). This experiment relied on repulsion forces generated by an electrical field to influence fibre diameter and bending instabilities at the same solution concentration, requiring strict parameter control. Incorporating cells into the mixture for precise 3D printing could simplify parameter setting, but the viability and proliferation of cells in silk-based bioink still need further investigation. It is anticipated to be a candidate ink material for 3D printing eardrum perforations.

However, challenges persist regarding the degradation of specific scaffold components and their long-term effects, requiring additional investigation.

There is a sensor mimicking the locust’s TM consisting of bisphenol-A ethoxylate dimethacrylate (BEMA) and BaTiO3 500 nm nanoparticles (NPs) via digital light processing stereolithography (Domingo-Roca et al., 2018). The manufacturing team observed that this sensor exhibited behaviour similar to the locust’s TM at each frequency. Despite differences in the mass distribution of specific regions on the locust TM and imperfections in handling interfaces during the printing process, which led to the opposite direction of wave propagation compared to the locust TM, the product still demonstrated the potential of 3D-printed TMs. Advancements in printing technology and precision facilitate the development of more biomimetic structures. However, for the bionic tympanic membrane intended for ear implantation, it is essential to consider its potential degradation by biological enzymes and how this degradation might impact its functionality. Future researchers should adapt and improve the material to address these concerns.

A biomimetic tissue consisting of epithelial and stromal components was laser-assisted printed to layer two types of stem cells within hydrogels. This approach mimics the stratified structure of the epithelial tissue in the human cornea, showcasing strong cell viability and differentiation (Sorkio et al., 2018). This biomimetic structure represents a pioneering demonstration of the feasibility of 3D bioprinting combined with human stem cells in corneal tissue engineering. It provides a solid foundation for bioprinting similar membranous structures, such as the TM, of which the whole three layers consist of cells and collagen less than 100 µm in average thickness.

## 6 Preclinical research studies and future clinical applications

In recent years, there have been ongoing explorations into the feasibility of using various materials or combining different technologies in 3D-printed scaffolds for treating TMP, yielding promising results. However, the safety and efficacy of many new repair materials still need to be clarified, necessitating evaluation before clinical trials. We outline various scaffold structures, bioink ingredients, printing methods, models used, outcome evaluations, and corresponding references in recent explorations of 3D-printed scaffolds for tympanic membrane repair (Table 1), detail the recent advancements in preclinical studies, including *in vitro* and animal models, that have laid the groundwork for future clinical applications.

Some studies have progressed in TM regeneration by utilising 3D bio-printed scaffolds for *in vivo* repair. A polycaprolactone/collagen/alginate-mesenchymal stem cell (PCAMSC) scaffold was developed and tested in a rat subacute perforation model (Jang et al., 2017). Following the repair of rat TM perforations using this scaffold compared to a scaffold without mesenchymal stem cells, it was found that the closure rate of perforations was higher in the experimental group with added stem cells than in the control group. Additionally, superior recovery of auditory brainstem response (ABR) thresholds and regenerated TM thickness was observed across all frequencies in the experimental group compared to the control group. Furthermore, the vibration velocity in the experimental group approached that of the standard control group. These findings suggest incorporating cells, drugs, or other bioactive substances can enhance TM healing and functional recovery. Optimising efficiency can be achieved by adjusting the types and quantities of drugs and cells loaded.

Patient-specific materials for TM repair could play a crucial role in facilitating the clinical implementation of 3D printing, particularly for preoperative preparation. Experimental evidence has already demonstrated that such materials can significantly reduce intraoperative time and even minimise trauma to autologous graft donor sites. Kuo et al. (2018) utilised endoscopic imaging and bioprinting to fabricate individually tailored TM grafts with GelMA and gelatin. The butterfly structure eliminates surgical glues and sutures; it enhances mechanical stability while including fibroblast components and accelerates wound healing. Animal studies have shown an improvement in healing TM perforations. In 2021, patient-specific 3D-printed templates were used in clinical surgery by the team led by Yang et al. (2021). They ingeniously leveraged the template obtained through the controllable nature of 3D printing paths to effectively guide cartilage-perichondrium cutting during surgery, significantly reducing the surgical time.

Moreover, the closure rate reached 100%, surpassing that of the non-template group. This shape better conforms to the patient’s physiological structure and provides a superior scaffold for TM healing. The template group’s postoperative air-bone gap (ABG) was significantly lower than the preoperative level.

Based on the findings from preclinical studies, we discover the potential clinical applications and benefits of 3D-printed tympanic membrane scaffolds. The previously mentioned scaffolds significantly accelerate wound healing, promote epithelial cell proliferation, and enhance fibre deposition. The controllable characteristics of 3D-printed filaments and the advantage of additive manufacturing with various bioinks for the same structure further enhance the benefits of 3D-printed scaffolds. Combined with clinical imaging and CAD modelling technologies, these scaffolds’ personalised and quantitative production is a critical advantage over other patch manufacturing methods. Although this technology requires interdisciplinary expertise for clinical translation and involves high time costs, its benefits outweigh the drawbacks. It remains an auspicious and innovative research direction. There is a need for further research to fully understand the long-term effects of these materials on hearing restoration. Additional research is needed to design and initiate clinical trials to evaluate the efficacy and safety of our 3D-printed scaffolds in human subjects.

## 7 Conclusion and future directions

3D printing for preparing materials for TM repair has garnered significant scholarly and popular attention. However, further comparative studies are necessary to understand the effects of different material properties, such as thicknesses and viscosities, on residual TM tissue. The impact of patches obtained through 3D printing on this tissue still needs to be fully understood compared to conventional patches. Notably, both mechanical properties and microstructure play crucial roles in prognosis. 3D printing enables control over fibre orientation in artificial TMs, albeit the disorganised yet ordered structure of the physiological TM.

For the bionic tympanic membrane intended for ear implantation, it is essential to consider its potential degradation by biological enzymes and how this degradation might impact its functionality. Future researchers should adapt and improve the material to address these concerns. Significant progress has recently been made in soft tissue additive manufacturing technologies. The development of multi-material 3D printing techniques has opened new possibilities for precisely fabricating soft tissues. By integrating the properties of different materials, researchers can achieve higher biocompatibility and mechanical performance. These advancements show great potential in various soft tissue manufacturing domains. As a member of the thin membranous tissues (TMT) (McLoughlin S. T. et al., 2023), current 3D printing research on the tympanic membrane is less extensive than studies on skin, cornea, and sclera. However, their experimental results provide valuable references for 3D printing of the tympanic membrane.

As previously mentioned, traditional extrusion printing methods have evolved to incorporate different extrusion forces to meet material structural requirements and improve resolution. The earliest inkjet printing has been innovatively modified into a direct cell printing method without material, solving the problem of cell damage during extrusion. Laser direct writing and other technological and bioink formula improvements have significantly advanced bioprinting. Combining the recent explorations in 3D printing for tympanic membrane repair listed in Table 1 further validates the application value of 3D printing technology in otology.

An ink with the appropriate composition is likely to create an optimal environment for fibroblast growth and fibrin alignment. The advantages of 3D printing, such as reduced damage to donor sites, decreased operative time, and the ability to control mechanical and biological properties and patch configuration, justify interdisciplinary clinical investigation.

A decade ago, the development of 3D printing and its integration with medical disciplines was unforeseen, but it has since made significant strides. There is potential to create personalised repair stents for acute and chronic TM perforations and produce patches that enhance hearing recovery. However, challenges remain in developing suitable composites and determining the optimal blend of synthetic and biomaterials, which necessitates approval from regulatory agencies and investment from commercial entities. Despite these challenges, the 3D printing of TMs has profoundly impacted the field of TM repair, expanding its horizons.

Applying 3D printing technology in tympanic membrane manufacturing has achieved significant results. Successful cases demonstrate its important value in otological surgeries. This technology enhances the precision and efficiency of tympanic membrane patch fabrication and reduces costs, providing a more economical and practical solution for clinical tympanic membrane repair. It simplifies the process of obtaining grafts during surgery, shortens operation time, and reduces the burden on patients. However, current research still faces several challenges, such as developing bioink formulations that can be rapidly translated to clinical use and conducting clinical trials. Future research should focus on addressing these issues to advance the field. Additionally, interdisciplinary collaboration should be encouraged to apply 3D printing technology to more areas of soft tissue manufacturing, promoting its widespread application in medical and industrial fields.
